# Albumin Redox Modifications Promote Cell Calcification Reflecting the Impact of Oxidative Status on Aortic Valve Disease and Atherosclerosis

**DOI:** 10.3390/antiox13010108

**Published:** 2024-01-16

**Authors:** Tamara Sastre-Oliva, Nerea Corbacho-Alonso, Elena Rodriguez-Sanchez, Elisa Mercado-García, Ines Perales-Sanchez, German Hernandez-Fernandez, Cristina Juarez-Alia, Teresa Tejerina, Luis F. López-Almodóvar, Luis R. Padial, Pedro L. Sánchez, Ernesto Martín-Núñez, Natalia López-Andrés, Gema Ruiz-Hurtado, Laura Mourino-Alvarez, Maria G. Barderas

**Affiliations:** 1 Department of Vascular Physiopathology, Hospital Nacional de Paraplejicos, Servicio de Salud de Castilla-La Mancha (SESCAM), 45071 Toledo, Spain; tsastre@sescam.jccm.es (T.S.-O.); ncorbacho@sescam.jccm.es (N.C.-A.); inesperalessanchez@gmail.com (I.P.-S.); ghernandezf@externas.sescam.jccm.es (G.H.-F.); cjuareza@externas.sescam.jccm.es (C.J.-A.); lmourino@sescam.jccm.es (L.M.-A.); 2 Department of Vascular Physiopathology, Hospital Nacional de Paraplejicos, Instituto de Investigación Sanitaria de Castilla-La Mancha (IDISCAM), 45071 Toledo, Spain; 3 Cardiorenal Translational Laboratory, Instituto de Investigación Imas12, Hospital Universitario 12 de Octubre, 28041 Madrid, Spainemercado.imas12@h12o.es (E.M.-G.); gemaruiz@h12o.es (G.R.-H.); 4 Department of Pharmacology, School of Medicine, Universidad Complutense de Madrid, 28040 Madrid, Spain; teje@med.ucm.es; 5 Cardiac Surgery, Hospital General Universitario de Toledo, Servicio de Salud de Castilla-La Mancha (SESCAM), 45007 Toledo, Spain; lopezalmodovar@yahoo.es; 6 Department of Cardiology, Hospital General Universitario de Toledo, Servicio de Salud de Castilla-La Mancha (SESCAM), 45007 Toledo, Spain; lrodriguez@sescam.org; 7 Department of Cardiology, Hospital Universitario de Salamanca-Instituto de Investigación Biomédica de Salamanca (IBSAL), University of Salamanca, 37007 Salamanca, Spain; plsanchez@saludcastillayleon.es; 8 Centro de Investigación Biomédica en Red de Enfermedades Cardiovasculares (CIBERCV), Instituto de Salud Carlos III, 28029 Madrid, Spain; 9 Cardiovascular Translational Research, Hospital Universitario de Navarra (HUN), Universidad Pública de Navarra (UPNA), 31008 Pamplona, Spain; emarnu87@gmail.com (E.M.-N.); natalia.lopez.andres@navarra.es (N.L.-A.); 10 Centro de Investigación Biomédica en Red de Enfermedades Cardiovasculares (CIBERCV), Hospital Universitario 12 de Octubre, 28041 Madrid, Spain

**Keywords:** aortic stenosis, interstitial cells, calcification, artery, aortic valve, oxidative stress, multimarker score

## Abstract

Calcific aortic valve disease (CAVD) and coronary artery disease (CAD) are related cardiovascular diseases in which common mechanisms lead to tissue calcification. Oxidative stress plays a key role in these diseases and there is also evidence that the redox state of serum albumin exerts a significant influence on these conditions. To further explore this issue, we used multimarker scores (OxyScore and AntioxyScore) to assess the global oxidative status in patients with CAVD, with and without CAD, also evaluating their plasma thiol levels. In addition, valvular interstitial cells were treated with reduced, oxidized, and native albumin to study how this protein and its modifications affect cell calcification. The differences we found suggest that oxidative status is distinct in CAVD and CAD, with differences in redox markers and thiol levels. Importantly, the in vitro interstitial cell model revealed that modified albumin affects cell calcification, accelerating this process. Hence, we show here the importance of the redox system in the development of CAVD, emphasizing the relevance of multimarker scores, while also offering evidence of how the redox state of albumin influences vascular calcification. These data highlight the relevance of understanding the overall redox processes involved in these diseases, opening the door to new studies on antioxidants as potential therapies for these patients.

## 1. Introduction

Calcific aortic valve disease (CAVD), also known as aortic valve (AV) stenosis, and coronary artery disease (CAD), are both progressive conditions with certain similarities, including common clinical risk factors [1]. The initial stages of CAVD and CAD share pathogenic mechanisms, including the endothelial dysfunction that favors inflammatory cell infiltration and lipid deposition in the tissues [2,3,4]. However, as AVs and arteries differ structurally and functionally, tissue stiffness has distinct consequences, and the most severe clinical manifestations in these structures have different causes. Indeed, the clinical manifestations in CAVD are due to obstructed blood flow, while in CAD, plaque stability is critical to avoid the release of prothrombotic agents [5,6].

Oxidative stress, which refers to an imbalance between antioxidant defenses and the production of reactive oxygen species (ROS), plays an important role in the calcification of vascular tissues, including AVs and arteries [7,8,9,10]. It is known that there are important differences between ROS production and the activity of certain enzymes in CAVD and CAD [11,12]. Moreover, when CAD is a comorbidity in CAVD patients, it is associated with alterations to the redox proteome, implying differences in their oxidative statuses [13]. Unfortunately, oxidative stress is not easy to assess due to its complex and multifactorial nature. Measuring individual biomarkers only partially defines the oxidative state, such that they may give rise to erroneous conclusions [14,15]. Hence, no single parameter can be recommended as a gold standard to determine an individual’s redox status. By contrast, global scores of oxidative status can be used that not only include markers of oxidative damage but also biomarkers of antioxidant defenses, offering a more complete overview of oxidative status [16]. Antioxidant capacity can be assessed by chemical-based assays that analyze free radical scavenging activity, such as 2,2′-azinobis (3-ethylbenzothiazoline-6-sulfonic acid) (ABTS), 2,2-Diphenyl-1-picrylhydrazylradical (DPPH) or Ferric Reducing Antioxidant Power (FRAP) assays [17,18,19]. Other methods, such as the measurement of the activity of low-molecular-weight antioxidants or antioxidants enzymes, are also usually used, especially in biological samples. This approach, combining individual plasma biomarkers of oxidative damage and antioxidant capacity to assess the overall oxidative balance, has been used successfully in several studies on different cardiovascular diseases (CVD) [14,20,21,22,23].

Considerable attention has been afforded to one potential biomarker of oxidative stress, the redox state of human serum albumin (HSA), a protein that is the most abundant source of thiol groups in human plasma [24]. Several studies have shown that the redox state of HSA is associated with a variety of health conditions, including CVD and type 2 Diabetes Mellitus (T2DM) [25,26]. Classically, the antioxidant activity of HSA has been attributed to the presence of the free sulfhydryl group of cysteine at position 34 (Cys34) [27]. Nevertheless, S-thiolation has also been described at different disulfide bonds of HSA [28]. Moreover, we recently found two new sites of oxidation and highlighted the implication of oxidized HSA for the development of CAVD in patients with CAD, maybe due to the transport of low-molecular-weight thiols inside the AV tissue [13]. Interestingly, endothelial cells cultured with oxidized albumin exhibit an increase in the markers of endothelial damage, as well as enhanced expression of inflammatory cytokines that is also related to vascular calcification [29]. However, the effects of oxidized albumin on valvular interstitial cells (VICs), the major cell population in heart valves, has not yet been studied.

**Figure 1:**
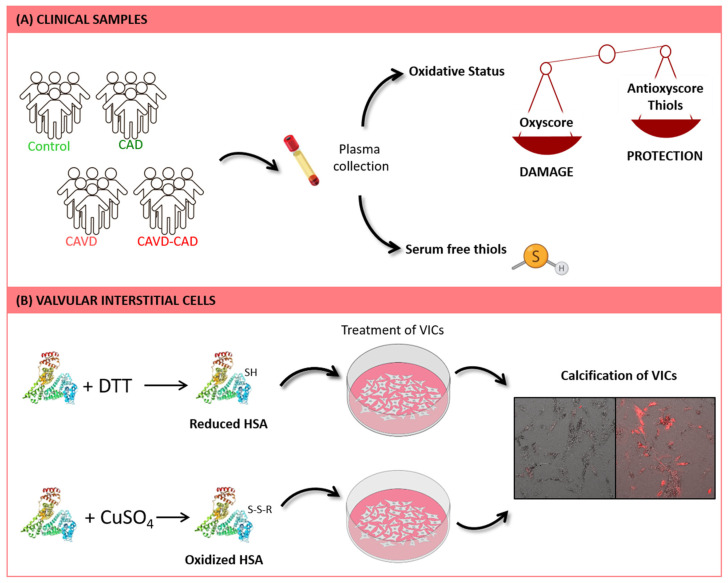
Workflow of the study. (**A**) Different markers of oxidative damage and antioxidant defense, including thiol levels, were measured in plasma samples from patients with CAVD, with and without CAD. (**B**) Human VICs were treated with oxidized, reduced, and native HSA. Subsequently, calcification of these cells was assessed by using Alizarin Red staining. Microscope images size is 665.6 µm × 665.6 µm.

Despite the known association between CAVD and CAD, the effects of the presence of CAD in CAVD patients in terms of global oxidative status have not been described. Here we measured different markers of oxidative damage and antioxidant defense, including the thiol levels in the plasma of patients, to gain a deeper understanding of the implication of HSA in these conditions. In addition, we also studied the effects of oxidized, reduced and native HSA on VICs in vitro, demonstrating its importance in the calcification process (Figure 1). The use of in vitro models is currently a cost-effective way to develop new pharmacological treatments. Moreover, understanding the global redox state associated with CAVD and CAD is key to taking a step forward towards precision personalized medicine, facilitating the prescription of adequate therapies to these patients.

## 2. Materials and Methods

### 2.1. Patient Selection

In this cross-sectional study, peripheral blood samples from 4 groups of subjects were collected: (i) controls without CAVD or CAD (C, n = 19); (ii) subjects with CAD alone (CAD, n = 16); (iii) patients with CAVD alone (CAVD, n = 20); and (iv) patients with both CAVD and CAD (CAVD + CAD, n = 20). All the patients were recruited from the Hospital General Universitario de Toledo (Spain).

Patients with any severe morbidity (e.g., ischemic heart disease with ventricular dysfunction or end-stage chronic kidney disease—CKD), any type of DM, bicuspid AV, a family or personal history of aortopathy, rheumatic valve disease or moderate or severe mitral valve disease were excluded from the study. Significant differences in terms of cardiovascular risk factors (gender, obesity, hypertension, and dyslipidemia) were avoided. The blood samples were collected in tubes containing EDTA and centrifuged for 10 min at 1125× *g*, and the resulting supernatant was immediately frozen at −80 °C until analysis. Collection, processing and storage were constant among the samples to avoid differences due to experimental handling.

This study was carried out in accordance with the guidelines of the Helsinki Declaration and the study design was approved by the local ethics committee. Signed informed consent was obtained from all individuals prior to their inclusion in the study.

### 2.2. Biomarkers of Oxidative Damage

Oxidative damage to proteins, lipids and DNA was evaluated by measuring protein carbonyls, oxidized low-density lipoprotein (oxLDL) and 8-hydroxy-20-deoxyguanosine (8-OHdG) levels. Protein carbonyl groups were measured using 2,4-dinitrophenylhydrazine in a protocol adapted to a microplate reader [30], and they were expressed as nmol/mg of total protein. OxLDL and 8-OHdG were assayed using commercial enzyme-linked immunosorbent assay (ELISA) kits (Mercodia AB, Uppsala, Sweden, and Stress-MarqBiosciences Inc., Victoria, BC, Canada, respectively), following the manufacturer’s instructions. Pro-oxidant xanthine oxidase (XOD) activity was determined with the Amplex Red assay (Invitrogen, Carlsbad, CA, USA) and expressed as mU/mg of total protein.

### 2.3. Biomarkers of Antioxidant Defense

Catalase and superoxide dismutase (SOD) activity was assessed as markers of enzymatic antioxidant activity. Plasma catalase activity was measured using the Amplex Red assay (Invitrogen) and expressed as U/mg of total protein. SOD activity was estimated using a colorimetric assay (Invitrogen, Carlsbad, CA, USA) and expressed as mU/mg of total protein. The overall activity of low-molecular-weight antioxidants or the total antioxidant capacity (TAC) was determined using an enhanced horseradish–peroxidase catalyzed-based luminol chemiluminescence assay adapted to a microplate reader [31]. Luminescence inhibition by the addition of plasma was used to calculate the area under the curve (AUC).

### 2.4. Serum Free Thiol Levels

Plasma thiol compounds were assayed using a SensoLyte^®^ Thiol Quantitation Assay Kit (AnaSpec, Fremont, CA, USA) according to the manufacturer’s instructions. This assay is based on the reaction of the sulfhydryl group of thiols with Ellman’s Reagent, generating 2-nitro-5-thiobenzoic acid (TNB) that in turn produces a yellow color that can be detected at 420 nm absorbance. The intensity of the color produced is proportional to the thiol concentration and it is expressed as µM thiol.

### 2.5. OxyScore and AntioxyScore

Multimarker scores of oxidative damage were computed as described previously [14,32,33,34]. Briefly, markers of oxidative damage or antioxidant defenses were standardized for each subject, using healthy subjects as a reference. The sum of the standardized values for protein carbonyls, oxLDL, 8-OHdG, and XOD activity was used to calculate the score of oxidative damage (OxyScore), whereas the sum of the standardized values of catalase and SOD activity and that of the TAC were used to calculate the score of global antioxidant defense (AntioxyScore).

### 2.6. Valvular Interstitial Cell Culture

Human cardiac VICs (Innoprot, Derio, Spain, P10462) were used in this study. These cells were isolated from heart valves and cryopreserved at passage one, after which they are guaranteed to expand further by 10 population doublings under the conditions indicated in the data sheet. VICs were cultured in Fibroblast Medium-2 (FM-2: Innoprot, Derio, Spain), designed for the optimal growth of normal human cardiac fibroblasts in vitro, a medium that contains essential and non-essential amino acids, vitamins, organic and inorganic compounds, hormones, growth factors, trace minerals, and a low concentration of fetal bovine serum (FBS, 5%). In these experiments, the cells were cultured for 7 days in two different media: (i) a special medium for fibroblasts (FIBm) that favors a quiescent phenotype (Hyclone Dulbecco’s Modified Eagle Medium (DMEM)), supplemented with 2% heat-inactivated FBS, 150 U/mL penicillin/streptomycin, 2 mM L-glutamine, 10 ng/mL fibroblast growth factor (FGF-2) and 50 ng/mL insulin and (ii) osteogenic medium (OSTm), to induce osteogenic differentiation of the HAVICs (FIBm supplemented with 50 µg/mL ascorbic acid, 10 mM β-glycerophosphate and 100 nM dexamethasone) [35,36]. As we observed differences in calcification after 24 h of culture (Figure S1), different concentrations of HSA were added to both these media and treatment was maintained during that period of time: either native HSA, OxHSA or RedHSA at a final concentration of 0.5, 1 and 2 mg/mL.

### 2.7. Preparation of Modified HSA

Oxidized HSA (OxHSA) was prepared following the protocol described previously, with minor modifications [37]. Briefly, HSA (Sigma Aldrich, St. Louis, MO, USA, Ref. A9731-5G) was diluted to 0.01 mM and incubated with CuSO_4_ (15 µmol/L) for 24 h at 37 °C before adding 0.15 mmol/L EDTA to prevent further oxidation. OxHSA was concentrated using regenerated cellulose membrane columns with a cutoff of 10 kDa, and the protein concentration was determined using a NanoDrop (Thermo Scientific, Waltham, MA, USA). The level of protein oxidation was determined with the OxyBlot Protein Oxidation Detection Kit, according to manufacturer’s specifications (Sigma Aldrich, St. Louis, MO, USA, Ref. S7150). Briefly, the OxyBlot kit derivatizes carbonyl groups to a 2,4-dinitrophenylhydrazone (DNP) moiety, which can then be detected with anti-DNP antibodies.

Albumin reduction was achieved as described previously, with minor modifications [29]. Diluted HSA (0.01 mM) was incubated with a 0.5 mM DTT solution for 1.5 h at 37 °C. At the end of the incubation, DTT was removed using regenerated cellulose membrane columns with a 10 kDa cutoff and the protein concentration was determined using a NanoDrop. To analyze protein reduction, reduced HSA (RedHSA) was labeled with SulfoBiotics PEG-PCMal (Dojindo Molecular Technologies Inc., Rockville, MD, USA, Ref. SB20-01), a 5 kDa Protein-SHifter, in accordance with the manufacturer’s instructions. Subsequently, RedHSA was analyzed by immunodetection as described previously [13].

### 2.8. Alizarin Red Staining

The cells were washed with PBS, fixed with 4% paraformaldehyde for 15 min, and then incubated for 10 min with Alizarin Red S (Sigma Aldrich, St. Louis, MO, USA) [38]. After washing with deionized water, calcium deposition was visualized under an Olympus IX83 inverted microscope, capturing 49 images per well, which were analyzed with ScanR software (v3.4.1., Olympus, Tokyo, Japan). Each of these experiments were performed in triplicate.

### 2.9. Statistical Analysis

Statistical analyses were performed using GraphPad Prism software (v.8.0.2., GraphPad Software Inc., San Diego, CA, USA) and SPSS software for Windows (v..15.0., SPSS Inc., Chicago, IL, USA). First, normality was assessed with the Kolmogorov–Smirnov test, and consequently, normally distributed variables were analyzed by parametric tests and those distributed non-normally were analyzed with non-parametric tests. Differences between the groups for the clinical parameters with discrete variables were calculated using a chi-squared test, while continuous variables such as oxidative markers were calculated by one-way ANOVA with a Bonferroni’s post hoc analysis for the four-group comparisons and adjusted for age and dyslipidemia. The descriptive data were presented as the mean ± standard deviation (SD) or as percentages.

## 3. Results

In this work, we initially analyzed clinical samples to determine the global oxidative statuses of four groups of subjects: controls without CAVD or CAD, subjects with CAD alone, patients with CAVD alone, or patients with both CAVD and CAD. We assessed markers of oxidative damage and of antioxidant defenses, as well as evaluating any reduction in thiol levels. In addition, we used an in vitro model to study the direct effect of the HSA redox state on the calcification of VICs.

### 3.1. Characteristics of the Study Population

The clinical characteristics of the study groups were compared (Table 1) and there were no significant differences between the groups in terms of the main cardiovascular risk factors, with the exception of the lower mean age of the controls when compared with the other groups. As dyslipidemia was almost significant, results are adjusted for these two factors: age and presence of dyslipidemia. Patients with DM were excluded from the study in order to minimize the presence of confounding factors.

### 3.2. Markers of Oxidative Damage and of Antioxidant Defenses

**Figure 2:**
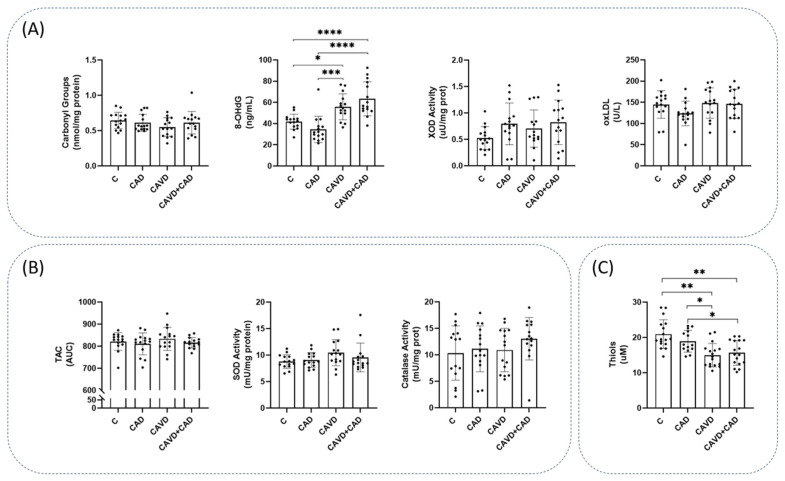
Markers of oxidative status in plasma from the four groups of study. (**A**) Markers of oxidative damage, including protein carbonyls, 8-hydroxy-2′-deoxyguanosine (8-OHdG) xanthine oxidase (XOD) activity and oxidized LDL (oxLDL) (n = 15 subjects/each group). (**B**) Markers of antioxidants defense, including total antioxidant capacity (TAC), superoxide dismutase (SOD) activity and catalase (CAT) activity (n = 15 subjects/each group). (**C**) Free reduced thiols (n = 14 subjects/CAD group and n = 17 subjects/C, CAVD and CAVD + CAD group). Data are represented as the mean ± SD. AUC, Area under the curve; C, Controls; CAD, Coronary artery disease; CAVD, Calcific aortic valve disease. * *p* < 0.05, ** *p* < 0.01, *** *p* < 0.001, **** *p* < 0.0001.

Oxidative damage to DNA was measured using the 8-OHdG levels, and it was significantly higher in patients with CAVD and CAD (63.34 ± 16.09 ng/mL) than in the controls (41.84 ± 7.07 ng/mL, adjusted *p*-value = 0.00) or patients with CAD alone (34.56 ± 12.41 ng/mL, adjusted *p*-value = 0.00: Figure 2A). There were also differences in this parameter between controls and patients with CAVD alone (adjusted *p*-value = 0.012: Figure 2A). Stronger pro-oxidant XOD enzymatic activity was detected in both groups of patients with CAD, although the differences between these subjects with and without CAVD were not significant (Figure 2A). In contrast, the protein carbonylation and oxLDL levels did not vary significantly between the three groups (Figure 2A). Similarly, catalase and SOD activity did not differ between the three groups of patients studied (Figure 2B), and along similar lines, the luminescence in the TAC assay was also similar in each group (Figure 2B). All mean values and the remaining statistical details are shown in Supplementary Tables S1 and S2.

### 3.3. Serum Thiol Levels

After measuring the thiols, we observed lower levels of reduced thiols in conjunction with the pathologies (controls = 20.94 ± 4.08 µM; CAD = 18.93 ± 3.05 µM; CAVD = 14.98 ± 3.31 µM; and CAVD + CAD = 15.63 ± 3.50 µM: Figure 2C). Significant differences existed between the controls and the CAVD patients, both without and with CAD (adjusted *p*-value < 0.01 in both cases), as well as between patients with CAD alone and patients with both pathologies (adjusted *p*-value < 0.05, see Supplementary Table S2 for the remaining statistical details).

### 3.4. Global Oxidative Status

**Figure 3:**
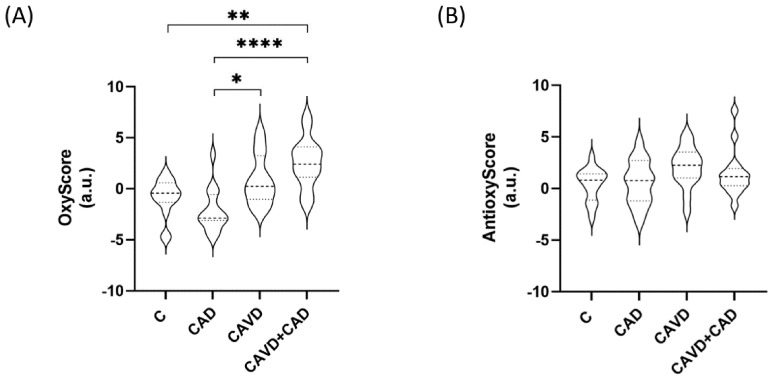
Multimarker scores of (**A**) oxidative damage (OxyScore) and (**B**) antioxidant defense (AntiOxyscore) (n = 15 subjects/each group). C, Controls; CAD, Coronary artery disease; CAVD, Calcific aortic valve disease. * *p* < 0.05, ** *p* < 0.01, **** *p* < 0.0001.

We found that the multimarker score of oxidative damage (OxyScore) was significantly higher in patients with CAD and CAVD (2.54 ± 2.41) than in the control patients (−0.87 ± 1.85, adjusted *p*-value = 0.001) or in patients with CAD alone (−2.15 ± 2.09, adjusted *p*-value = 0.000). By contrast, there were no differences in the multimarker antioxidant defense scores (AntioxyScore) of the groups (Figure 3, see Supplementary Tables S1 and S2 for all the mean values and the remaining statistical details).

### 3.5. Cell Calcification on Exposure to HSA

**Figure 4:**
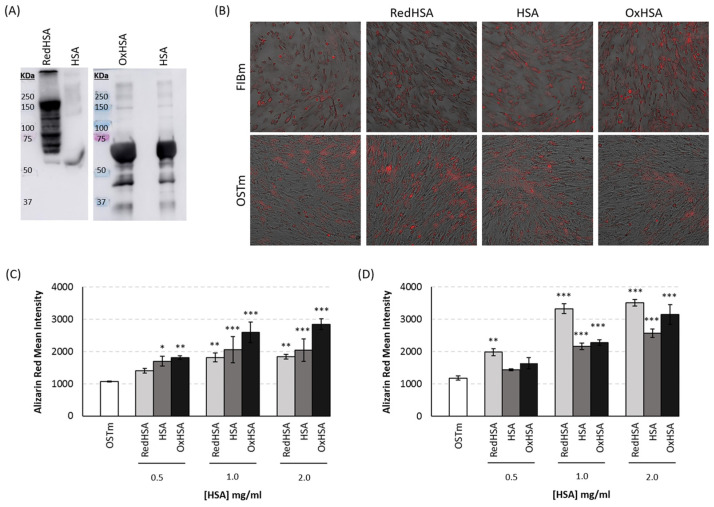
Results from in vitro model. (**A**) Immunoblot of modified HSA. Reduced HSA (RedHSA) was labeled with SulfoBiotics-PEG-PCMal, which produces a mobility shift corresponding to approximately 5 kDa for each molecule of PEG-PCMal bound to a free thiol group of the target protein. Thus, after the blotting, several bands corresponding to RedHSA are observed (from 60 to 150 kDa), while the lane of native HSA only shows one band. Oxidized HSA (OxHSA) was evaluated using OxyBlot Protein Oxidation Detection Kit. This kit allows the immunodetection of carbonyl groups through DNP-derivatization and subsequent detection of this DNP moiety with a specific primary antibody. It can be observed that the bands corresponding to OxHSA are more intense than the bands corresponding to native HSA. (**B**) Representative images of the Alizarin Red staining at a concentration of 1 mg/mL of modified or native HSA after 24 h of treatment. Microscope images size is 665.6 µm × 665.6 µm. (**C**) Calcification levels of cells cultured for 24 h in medium for fibroblast (FIBm) when supplemented with different concentrations of modified or native HSA. (**D**) Calcification levels of cells cultured for 24 h in osteogenic medium (OSTm) when supplemented with different concentrations of modified or native HAS was measured using Alizarin red staining. All experiments were performed in triplicate. * *p* < 0.05, ** *p* < 0.01, *** *p* < 0.001.

Human VICs were cultured in the presence of different concentrations of HSA, including basal, RedHSA and OxHSA. HSA modifications were confirmed by immunoblot (Figure 4A). After 24 h in culture with FIBm or OSTm, differences in calcification were evident, and specifically, the calcification of cells cultured in FIBm augmented when exposed to higher concentrations of OxHSA (Figure 4B,C: *p*-values < 0.005 in all cases). Surprisingly, cells respond in a different manner to HSA when cultured in FIBm or OSTm. In OSTm, the cells appeared to calcify more in the presence of high concentrations of RedHSA (Figure 4B,D), with significant differences when compared to the cells maintained in OSTm alone (*p*-values < 0.005 for the three concentrations, the mean values and the remaining statistical details can be found in Supplementary Tables S3 and S4).

## 4. Discussion

Oxidative stress represents an imbalance between the production of ROS and the ability to detoxify reactive products and/or repair the resulting damage. The toxic effects of oxidative stress caused by peroxide and free radical production produce damage in all cell components, affecting proteins, lipids and DNA. Despite the importance of oxidative stress in biology and medicine, it is a phenomenon that is still challenging to measure. ROS are highly reactive, and consequently, they have short half-lives in biological environments, making them difficult to measure directly. Indeed, even indirect estimates of their abundance and reactivity are complicated. As such, a common method to assess oxidative stress is to measure the macromolecules oxidized (lipids, proteins, and DNA) and the antioxidants (enzymatic and non-enzymatic antioxidants) [39,40].

Oxidative stress is implicated in pathophysiological vascular calcification, and it plays a significant role in the development and progression of CAVD and CAD, even being able to predict cardiovascular events [41,42,43,44,45,46]. Given this association, we evaluated here the global oxidative status associated with both these pathologies using the multimarker parameters, OxyScore and AntioxyScore, as well as the plasma thiol levels. In addition, the effect of the redox state of HSA on VICs in culture was also studied.

### 4.1. Oxidative Status

Higher levels of 8-OHdG were found in CAVD patients relative to the controls, which indicates more severe oxidative damage to DNA. Higher levels of 8-OHdG were associated with an unfavorable 30-day prognosis following AV replacement and better discrimination of standard clinical models predicting the 30-day and 1-year risk of standardized end-points [47,48]. Indeed, we found higher levels of 8-OHdG in patients with both CAVD and CAD, consistent with the fact that CAD is a negative predictive indicator in patients with CAVD [49,50,51]. It is important to highlight here that the differences between patients with and without CAD are not significant, although a clear tendency does exist. Previous studies showed the importance of 8-OHdG in the atherosclerotic process, and high levels of 8-OHdG have been found in fragments of aorta from patients with severe atherosclerotic lesions. In addition, 8-OHdG levels have been correlated with the number of vessels affected [52,53] and, in leukocyte mtDNA in diabetic patients, with coronary stenosis severity [54]. An extended meta-analysis of 14 studies showed that the association between 8-OHdG levels and CVD is largely independent of diabetes, hyperlipidemia, body mass index and smoking habits. By contrast, the association between 8-OHdG levels and CVD was stronger in younger subjects, and a higher prevalence of hypertension was associated with smaller differences in 8-OHdG levels between CVD patients and controls [55]. This phenomenon might be explained by the reduced DNA turnover associated with aging [56] and by the higher levels of 8-OHdG found in hypertensive subjects relative to normotensive controls [57]. The use of ELISA kits for the measurement of 8-OHdG should also be highlighted. Although the gold standard for 8-OHdG is high performance liquid chromatography coupled with an electrochemical detector, immunological methods, such as ELISA, are less costly and time consuming than chromatography. Furthermore, ELISA kits are tools widely used in clinical practice as they are used in surveillance and disease monitoring as well as diagnostic tools. The use of these methodologies at these stages of the research is a step forward in looking for clinical application and facilitates its implementation. It is important to note that the cohort of patients with severe CAVD studied here has an average age above 75 years old. Moreover, hypertension is more prevalent in patients with CAVD, such that we cannot rule out this comorbidity from the study [58]. To overcome this issue, we avoided significant differences in hypertension among the study groups. For those reasons, the differences we found in 8-OHdG levels may be less pronounced than those described elsewhere.

Although we do not find more significant differences in markers of oxidative damage, by focusing on XOD activity, a non-significant increase in ROS was evident in CAD patients relative to non-CAD patients. This enzyme is implicated in ROS production [59], and it is believed to promote inflammatory responses and atherosclerotic plaque formation [60], consistent with the enhanced activity found in these patients.

Regarding antioxidant levels, it has been previously described that expression and activity of antioxidant enzymes are reduced in calcified regions of stenotic AV when compared with non-calcified regions. Additionally, although oxidative stress appears to be increased in stenotic AV, the mechanisms that account for this oxidative stress differ greatly from those observed in atherosclerotic arteries [11]. Supporting our data from previous studies, our results showed no differences in individual markers of antioxidant defense [14].

Importantly, we found fewer reduced thiol groups in patients with CAVD, groups that offer protection against oxidative stress through ROS scavenging. In fact, low levels of serum free thiols are associated with a higher risk of cardiovascular events [61]. Recently, an analysis of four cohorts from different European countries observed associations of both high derivatives of reactive oxygen metabolites levels and low total thiol levels with fatal myocardial infarction and stroke, two atherosclerotic diseases [62]. The results suggest an important contribution of an imbalanced redox system to the etiology of mainly fatal MI and stroke events. Indeed, the results indicated that thiol levels may be an important factor in the development and progression of CVD, including AV disease in the context of atherosclerosis, as we hypothesized previously [63].

In considering the global scores, we observed a gradual increase in the OxyScore of patients with CAVD, both with and without CAD, relative to the controls. By contrast, the AntioxyScore does not vary significantly. There is evidence that oxidative stress may activate antioxidant defense mechanisms, for example through the activity of transcription factors like Nrf2 [64]. However, other studies showed that an excessive amount of ROS will produce an impairment in the antioxidant system, dampening the activity of antioxidant enzymes [65]. We previously found that despite an increase in oxidative end-products, patients with CAVD and T2DM do not respond to oxidative insults [14], as evident here. In fact, patients with CAVD and CAD have higher OxyScore and lower AntiOxyscore values than patients with CAVD alone, although these differences were not significant. In the light of this, we hypothesized that these patients have weaker cellular resilience to oxidative stressors, and as such, they are unable to counteract the harmful effects of ROS through such mechanisms.

### 4.2. How the Human Serum Albumin Redox State Affects Valvular Interstitial Cells

As we have discussed above, patients with CAVD have lower levels of reduced thiol groups than controls, i.e., a lower capacity for protection against ROS. This result highlights the importance of HSA in both CAVD and CAD, as this protein plays a key role in the antioxidative capacity of blood plasma and its ability to manage ROS [66] given that it is responsible for trapping more than 70% of the free radicals in plasma [67]. However, more studies will be needed to fully understand the role of thiols and HSA in CAVD and CAD.

**Figure 5:**
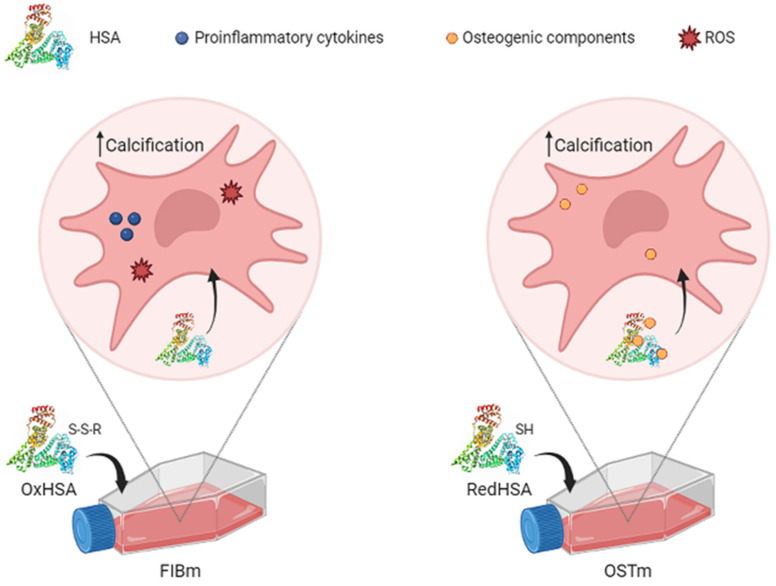
Hypothesis about the effect of oxidized HSA (OxHSA) in medium for fibroblasts (FIBm) and reduced HSA (RedHSA) in osteogenic medium (OSTm). In both cases, an intense calcification is observed in valvular interstitial cells, but OxHSA is believed to induce proinflammatory cytokines and reactive oxygen species, while RedHSA may act as a carrier of the osteogenic components that have the OSTm.

The redox state of HSA has been associated with greater cardiovascular risk in patients with other comorbidities, such as CKD or COVID-19 infection [68,69,70]. Moreover, patients with CAVD and CAD have more circulating OxHSA in their plasma [13]. Here, to assess if this circulating HSA may be harmful to VICs, we studied the effects of different concentrations of modified HSA in vitro. As a result, calcification was observed to be affected by exposure to RedHSA and OxHSA, although interestingly, differences were found when HSA was added to FIBm or OSTm. Cell calcification is stronger when FIBm is supplemented with OxHSA, consistent with the previous hypothesis that oxidized circulating HSA might act as a carrier of low molecular weight thiols, which could in turn be released into the subendothelial space and aggravate the oxidative stress suffered by local tissues [13]. In other cell populations, such as endothelial cells or neutrophils, OxHSA produces an increase in apoptosis, inflammation, senescence and intracellular ROS production, thereby enhancing oxidative stress and cell damage [37,71] (Figure 5). Importantly, we also found enhanced calcification with non-modified HSA, which makes sense, as it usually undergoes some degree of oxidation. Alternatively, calcification is more pronounced in OSTm supplemented with RedHSA, the antioxidant activity of which is known to be stronger than that of OxHSA [72]. The binding properties of HSA depend on the three-dimensional structure of the binding sites, a structure that is altered by redox modifications [73]. Due to these structural changes, the redox state influences the drug binding properties of HSA. Thus, we hypothesize that RedHSA has a stronger affinity for the osteogenic components of OSTm (ascorbic acid, β-glycerophosphate, and dexamethasone), increasing the uptake of these substances by VICs (Figure 4). This would explain why these cells calcified in a more intense manner. If we consider the important role of has as a drug carrier, this result could be key in the clinical field for therapeutic purposes.

Here, we obtain evidence of the importance of the redox system in the development of CAVD. We found that in patients with both CAVD and CAD, CAVD has a stronger effect on the oxidative status than CAD. There has been considerable interest in antioxidants in recent years for their protective role in CVD, even in conjunction with CAVD and CAD, and through different mechanisms such as NADPH Oxidase 2 inhibition [42,74,75]. Better understanding of which redox processes are affected in these conditions will be essential to selecting the most adequate therapy for each patient. The application of multimarker scores may play a significant role in selecting such therapies, as they provide information about the different mechanisms that are implicated in maintaining the redox balance. In addition, in vitro models are important to understand the effects of different substances on specific tissues. We demonstrate that the redox state of HSA influences calcification, and we have evidence of differential transport rates according to that state. It is important to highlight the importance of this kind of study to assess the influence of different mediators of oxidative stress. These molecules have a physiological role, and in many cases, the therapeutic goals of non-targeted broad-spectrum antioxidants fail [76]. For that reason, such studies must adopt a precision medicine perspective. Indeed, understanding the specific redox changes that occur in patients with CAVD and different comorbidities, such as CAD, is essential, as patients with different comorbidities could have an additive accumulation of redox biomarkers [77]. These studies will pave the way for the design of new therapeutic strategies that may slow down the course of the disease, such as through the development of targeted drugs.

The main limitation of this study is that the sample size included is relatively small, as well as the heterogeneity in age. Although we excluded patients with DM, it was impossible to avoid other risk factors, such as hypertension, due to its elevated prevalence in patients with developed CVD. Thus, further studies on larger cohorts will be necessary to obtain more information about the effects of these comorbidities and to achieve better patient stratification based on their clinical characteristics. Importantly, different cardiovascular drugs, such as statins, have antioxidant properties that may affect outcomes. Unfortunately, information about patient medication is lacking, which is also a limitation of this study. Furthermore, additional in vitro studies should be performed to characterize the different structural modifications albumin undergoes.

## 5. Conclusions

Here, we provide information about the redox alterations found in patients with CAVD and CAD, which indicates that these impair the antioxidant responses in these patients such that they fail to fully neutralize the harmful effects of ROS. In addition, we demonstrate that different HSA redox states affect valvular cell calcification in different manners, potentially accelerating this process. These results highlight the importance of understanding the whole redox processes taking place in these diseases, as these mechanisms differ in each pathology. As such, future studies might focus on antioxidants as personalized therapies for these patients from a precision medicine perspective.

## Figures and Tables

**Table 1:** Clinical characteristics of the subjects studied: C, control without calcific aortic valve disease or coronary artery disease; CAD, control with coronary artery disease; CAVD, calcific aortic valve disease without coronary artery disease; CAD, calcific aortic valve disease with coronary artery disease. Abbreviations: M/F, male/female; AHT, Arterial Hypertension; *p*, *p*-value.

Clinical Characteristics	C (n = 19)	CAD (n = 17)	CAVD (n = 20)	CAVD + CAD (n = 20)	*p*
Age	65.4 ± 8.5	80.81 ± 9.2	80.70 ± 6.1	76.2 ± 7.8	0.000
Gender (M/F)	10/9	9/7	11/9	14/6	0.684
%AHT	47	62	80	55	0.183
%Dyslipidemia	26	62	45	65	0.064
%Diabetes	0	0	0	0	1.000
%Smokers	15	0	0	10	0.140

## Data Availability

Data are contained within the article and supplementary material.

## Supplementary Materials

The following supporting information can be downloaded at: https://www.mdpi.com/article/10.3390/antiox13010108/s1, Figure S1. Calcification levels of cells cultured for 7 days in medium for fibroblast (FIBm) and osteogenic medium (OSTm). Results show significant differences in calcification after 24 h of culture. ** *p* < 0.01, *** *p* < 0.001; Table S1. Antioxidant defense markers of oxidative damage (the mean ± SD is shown); Table S2. Comparison of antioxidant defense markers and markers of oxidative damage between the different groups studied (age and dyslipidemia-adjusted *p*-values are shown); Table S3. Calcification of VICs (the mean intensity of Alizarin Red staining is shown): FIBm, medium for fibroblast medium; HSA, human serum albumin; OSTm, osteogenic medium; OxHSA, oxidized human serum albumin; RedHSA, reduced human serum albumin; Table S4. Comparison of the treatments of VICs in vitro (*p*-values are shown): FIBm, medium for fibroblast medium; HSA, human serum albumin; OSTm, osteogenic medium; OxHSA, oxidized human serum albumin; RedHSA, reduced human serum albumin. The *p*-values < 0.05 are shown in bold and italics.
