# Supplementary material for: Albumin Redox Modifications Promote Cell Calcification Reflecting the Impact of Oxidative Status on Aortic Valve Disease and Atherosclerosis
Source: Antioxidants (Basel). 2024 Jan 16;13(1):108. doi: 10.3390/antiox13010108 (PMC10812654; doi:10.3390/antiox13010108)

Supplementary Material

Albumin Redox Modifications Promote Cell Calcification Reflecting the impact of Oxidative Status on Aortic Valve Disease and Atherosclerosis

Tamara Sastre-Oliva[1, 2†], Nerea Corbacho-Alonso[1,2†], Elena Rodriguez-Sanchez[3], Elisa Mercado-García[3], Ines Perales-Sanchez[1,2], German Hernandez-Fernandez[1,2], Cristina Juarez-Alia[1,2], Teresa Tejerina[4], Luis F. López-Almodóvar[5], Luis R. Padial[6], Pedro L Sánchez[7,8], Ernesto Martín-Núñez[9], Natalia López-Andrés[9], Gema Ruiz-Hurtado[3, 10], Laura Mourino-Alvarez[1,2‡], Maria G Barderas[1,2‡]

† These authors contributed equally to this work.
‡ These authors contributed equally to this work.
Correspondence: M.G. Barderas, megonzalezb@sescam.jccm.es; marugbarderas@gmail.com; Phone: +34 925247826 FAX: +34 925247745.

Tables

Table S1. Antioxidant defense markers of oxidative damage (the mean ± SD is shown).

	C	C+CAD	CAVD	CAVD+CAD
**Carbonyls (nmol/mg protein)**	0.64±0.11	0.61±0.12	0.55±0.13	0.61±0.16
**oxLDL (U/L)**	144.78±32.31	123.7±29.08	148.18±35.81	146.48±34.53
**80HdG (ng/mL)**	41.84±7.07	34.56±12.41	55.82±12.24	63.34±16.09
**XOD (μU/mg protein)**	0.52±0.22	0.79±0.4	0.71±0.35	0.82±0.42
**TAC (AUC)**	821.27±40.52	810.53±49.38	833.27±52.59	815.77±23.63
**CAT (mU/mg protein)**	10.35±5.12	11.14±4.33	10.9±4.08	13.03±4.01
**SOD (mU/mg protein)**	8.79±1.29	9.08±1.39	10.44±2.46	9.57±2.71
**Thiols (μM)**	20.94±4.08	18.93±3.05	14.98±3.31	15.63±3.50
**OxyScore (a.u.)**	-0.87±1.85	-2.15±2.09	0.87±2.46	2.54±2.41
**AntioxyScore (a.u.)**	0.42±1.59	0.56±2.15	2.06±1.97	1.43±2.27

Table S2. Comparison of antioxidant defense markers and markers of oxidative damage of between the different groups studied (age and dyslipidemia-adjusted p-values are shown).

		Carbonyls	OxLDL	80HdG	XOD	TAC	CAT	SOD	Thiol	OxyScore	AntioxyScore
**C vs**	**CAD**	1.000	1.000	1.000	0.811	1.000	1.000	1.000	0.505	1.000	1.000
	**CAVD**	0.203	1.000	0.012	1.000	1.000	1.000	1.000	0.002	0.370	1.000
	**CAVD+CAD**	1.000	1.000	0.000	0.358	1.000	1.000	1.000	0.003	0.001	1.000
**CAD vs**	**C**	1.000	1.000	1.000	0.811	1.000	1.000	1.000	0.505	1.000	1.000
	**CAVD**	1.000	0.377	0.000	1.000	0.796	1.000	0.576	0.026	0.010	0.250
	**CAVD+CAD**	1.000	0.438	0.000	1.000	1.000	1.000	1.000	0.049	0.000	1.000
**CAVD vs**	**C**	0.203	1.000	0.012	1.000	1.000	1.000	1.000	0.002	0.370	1.000
	**CAD**	1.000	0.377	0.000	1.000	0.796	1.000	0.576	0.026	0.010	0.250
	**CAVD+CAD**	1.000	1.000	0.404	1.000	1.000	1.000	1.000	1.000	0.130	1.000
**CAVD+CAD vs**	**C**	1.000	1.000	0.000	0.358	1.000	1.000	1.000	0.003	0.001	1.000
	**CAD**	1.000	0.438	0.000	1.000	1.000	1.000	1.000	0.049	0.000	1.000
	**CAVD**	1.000	1.000	0.404	1.000	1.000	1.000	1.000	1.000	0.130	1.000

Table S3. Calcification of VICs (the mean intensity of Alizarin Red stainig is shown): FIBm, medium for fibroblast medium; HSA, human serum albumin; OSTm, osteogenic medium; OxHSA, oxidized human serum albumin; RedHSA, reduced human serum albumin.

[HSA]		FIBm	OSTm
0 mg/ml	Basal medium	1069.36±7.83	1180.61±58.63
0.5 mg/ml	RedHSA	1400.52±68.45	1981.23±104.15
	HSA	1699.98±157.90	1438.09±29.40
	OxHSA	1804.57±60.09	1642.35±174.97
1 mg/ml	RedHSA	1811.12±138.81	3324.85±151.09
	HSA	2052.98±402.16	2160.24±104.73
	OxHSA	2591.99±319.68	2272.07±85.93
2 mg/ml	RedHSA	1839.98±68.89	3510.40±106.34
	HSA	2044.81±349.25	2560.01±130.42
	OxHSA	2844.12±162.84	3145.17±307.82

Table S4. Comparison of the treatments of VICs in vitro (p-values are shown): FIBm, medium for fibroblast medium; HSA, human serum albumin; OSTm, osteogenic medium; OxHSA, oxidized human serum albumin; RedHSA, reduced human serum albumin. The P-values<0.05 are shown in bold and italics.

		FIB										OST									
		FIBm	HSA (0.5)	HSA (1)	HSA (2)	OxyHSA (0.5)	OxyHSA (1)	OxyHSA (2)	RedHSA (0.5)	RedHSA (1)	RedHSA (2)	OSTm	HSA (0.5)	HSA (1)	HSA (2)	OxyHSA (0.5)	OxyHSA (1)	OxyHSA (2)	RedHSA (0.5)	RedHSA (1)	RedHSA (2)
FIBm	FIBm		***0.028***	***0.000***	***0.000***	***0.003***	***0.000***	***0.000***	1.000	***0.003***	***0.002***	1.000	1.000	***0.000***	***0.000***	0.090	***0.000***	***0.000***	***0.000***	***0.000***	***0.000***
	HSA (0.5)	***0.028***		1.000	1.000	1.000	***0.000***	***0.000***	1.000	1.000	1.000	0.253	1.000	0.754	***0.000***	1.000	0.092	***0.000***	1.000	***0.000***	***0.000***
	HSA (1)	***0.000***	1.000		1.000	1.000	0.174	***0.001***	***0.018***	1.000	1.000	***0.000***	***0.039***	1.000	0.320	1.000	1.000	***0.000***	1.000	***0.000***	***0.000***
	HSA (2)	***0.000***	1.000	1.000		1.000	0.149	***0.001***	***0.022***	1.000	1.000	***0.000***	***0.046***	1.000	0.274	1.000	1.000	***0.000***	1.000	***0.000***	***0.000***
	OxyHSA (0.5)	***0.003***	1.000	1.000	1.000		***0.001***	***0.000***	1.000	1.000	1.000	***0.033***	1.000	1.000	***0.002***	1.000	0.662	***0.000***	1.000	***0.000***	***0.000***
	OxyHSA (1)	***0.000***	***0.000***	0.174	0.149	***0.001***		1.000	***0.000***	***0.001***	***0.002***	***0.000***	***0.000***	1.000	1.000	***0.000***	1.000	0.133	***0.042***	***0.003***	***0.000***
	OxyHSA (2)	***0.000***	***0.000***	***0.001***	***0.001***	***0.000***	1.000		***0.000***	***0.000***	***0.000***	***0.000***	***0.000***	***0.010***	1.000	***0.000***	0.092	1.000	***0.000***	0.520	***0.014***
	RedHSA (0.5)	1.000	1.000	***0.018***	***0.022***	1.000	***0.000***	***0.000***		1.000	1.000	1.000	1.000	***0.002***	***0.000***	1.000	***0.000***	***0.000***	0.077	***0.000***	***0.000***
	RedHSA (1)	***0.003***	1.000	1.000	1.000	1.000	***0.001***	***0.000***	1.000		1.000	***0.029***	1.000	1.000	***0.002***	1.000	0.744	***0.000***	1.000	***0.000***	***0.000***
	RedHSA (2)	***0.002***	1.000	1.000	1.000	1.000	***0.002***	***0.000***	1.000	1.000		***0.016***	1.000	1.000	***0.004***	1.000	1.000	***0.000***	1.000	***0.000***	***0.000***
OSTm	OSTm	1.000	0.253	***0.000***	***0.000***	***0.033***	***0.000***	***0.000***	1.000	***0.029***	***0.016***		1.000	***0.000***	***0.000***	0.734	***0.000***	***0.000***	***0.001***	***0.000***	***0.000***
	HSA (0.5)	1.000	1.000	***0.039***	***0.046***	1.000	***0.000***	***0.000***	1.000	1.000	1.000	1.000		***0.004***	***0.000***	1.000	***0.000***	***0.000***	0.161	***0.000***	***0.000***
	HSA (1)	***0.000***	0.754	1.000	1.000	1.000	1.000	***0.010***	***0.002***	1.000	1.000	***0.000***	***0.004***		1.000	0.261	1.000	***0.000***	1.000	***0.000***	***0.000***
	HSA (2)	***0.000***	***0.000***	0.320	0.274	***0.002***	1.000	1.000	***0.000***	***0.002***	***0.004***	***0.000***	***0.000***	1.000		***0.000***	1.000	0.071	0.080	***0.002***	***0.000***
	OxyHSA (0.5)	0.090	1.000	1.000	1.000	1.000	***0.000***	***0.000***	1.000	1.000	1.000	0.734	1.000	0.261	***0.000***		***0.029***	***0.000***	1.000	***0.000***	***0.000***
	OxyHSA (1)	***0.000***	0.092	1.000	1.000	0.662	1.000	0.092	***0.000***	0.744	1.000	***0.000***	***0.000***	1.000	1.000	***0.029***		***0.000***	1.000	***0.000***	***0.000***
	OxyHSA (2)	***0.000***	***0.000***	***0.000***	***0.000***	***0.000***	0.133	1.000	***0.000***	***0.000***	***0.000***	***0.000***	***0.000***	***0.000***	0.071	***0.000***	***0.000***		***0.000***	1.000	1.000
	RedHSA (0.5)	***0.000***	1.000	1.000	1.000	1.000	***0.042***	***0.000***	0.077	1.000	1.000	***0.001***	0.161	1.000	0.080	1.000	1.000	***0.000***		***0.000***	***0.000***
	RedHSA (1)	***0.000***	***0.000***	***0.000***	***0.000***	***0.000***	***0.003***	0.520	***0.000***	***0.000***	***0.000***	***0.000***	***0.000***	***0.000***	***0.002***	***0.000***	***0.000***	1.000	***0.000***		1.000
	RedHSA (2)	***0.000***	***0.000***	***0.000***	***0.000***	***0.000***	***0.000***	***0.014***	***0.000***	***0.000***	***0.000***	***0.000***	***0.000***	***0.000***	***0.000***	***0.000***	***0.000***	1.000	***0.000***	1.000	

Figures

Figure S1. Calcification levels of cells cultured for 7 days in medium for fibroblast (FIBm) and osteogenic medium (OSTm). Results show significant differences in calcification after 24 h of culture. ** $p < 0.01$, *** $p < 0.001$.

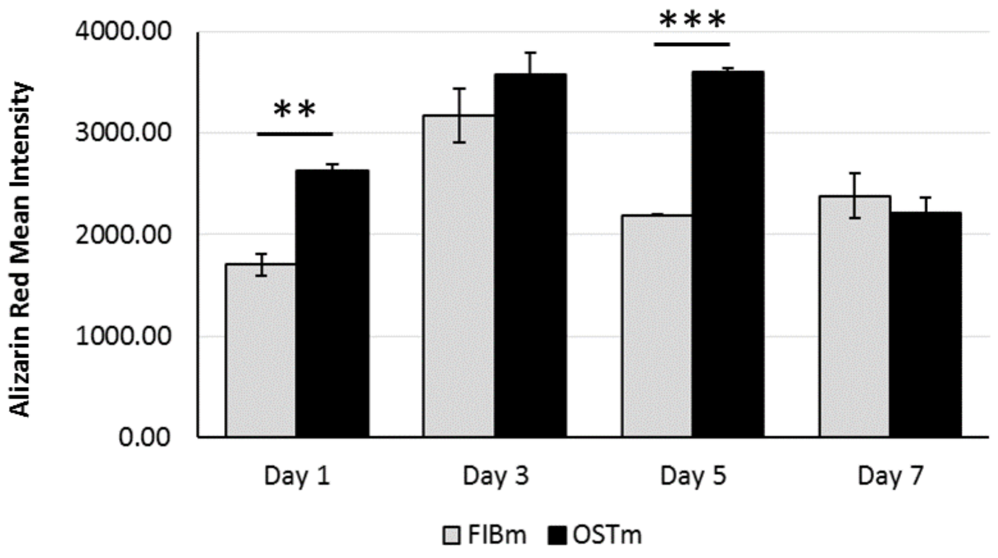

Supplement: Supplementary file 1 [file antioxidants-13-00108-s001.zip › antioxidants-2793700-supplementary.pdf]
